# Transversus abdominis plane block for an emergency laparotomy in a high-risk, elderly patient

**DOI:** 10.4103/0019-5049.65377

**Published:** 2010

**Authors:** Surekha S Patil, Shonali C Pawar, VM Divekar, Rochana G Bakhshi

**Affiliations:** Department of Anaesthesiology, Padmashree Dr. D.Y. Patil Medical College and Hospital Research Centre, Nerul, Navi-Mumbai, India

**Keywords:** Laparotomy, Petit triangle, transversus abdominis plane block

## Abstract

A 72-year-old male patient with gall bladder perforation and small intestinal obstruction from impacted gall stone was posted for emergency laparotomy. He had congestive heart failure, severe hypertension at admission and history of multiple other coexisting diseases. On admission, he developed pulmonary oedema from systolic hypertension which was controlled by ventilatory support, nitroglycerine and furosemide. Preoperative international normalized ratio was 2.34 and left ventricular ejection fraction was only 20%. Because of risk of exaggerated fall in blood pressure during induction of anaesthesia (general or neuraxial), a transversus abdominis plane block via combined Petit triangle and subcostal technique was administered and supplemented with Propofol sedation.

## INTRODUCTION

**Figure 1:**
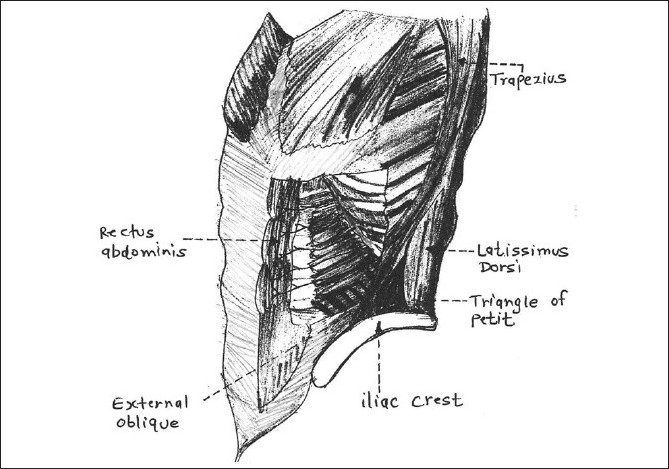
Triangle of Petit bounded by Latissimus dorsi muscle posteriorly, external oblique anteriorly and iliac crest forming base

Patients with coronary artery disease with congestive heart failure and multiple coexisting diseases posted for major abdominal surgery are unquestionably at a high risk for perioperative complications.[1] Regional anaesthesia is an attractive option in conditions where risk of perioperative mortality is very high, especially in elderly patients. Transversus abdominis plane (TAP) block given by Petit triangle approach has been used effectively for intra- and postoperative analgesia for abdominal surgeries [Figure 1].[2] There are recent reports of ultrasound-guided approach and subcostal technique for TAP block for abdominal surgeries above the umbilicus.[3] We herein report a high-risk American Society of Anaesthesiologists (ASA) IV physical status, elderly patient who successfully underwent emergency abdominal surgery under TAP block using a combined Petit triangle and subcostal technique supplemented with intravenous sedation. We also discuss how our compromised cardiac status patient did well with TAP block both intraoperatively and postoperatively.

## CASE REPORT

**Figure 2:**
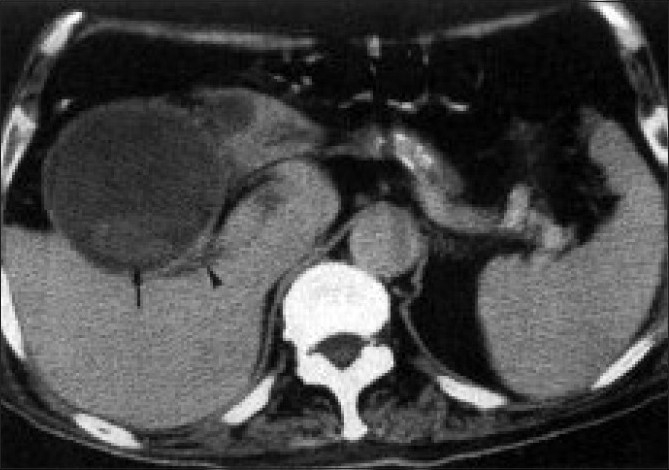
Contrast CT scan showing gallstones (arrow) with gallbladder rupture and fluid localized at the gallbladder fossa (arrowhead), penetrating into the left subhepatic area.

A 72-year-old, 85-kg male was brought to the emergency room with abdominal pain, vomiting and constipation. He had a past medical history of severe hypertension, ischemic heart disease, diabetes mellitus, peripheral vascular disease, cerebrovascular accident (right hemiplegia), and deep vein thrombosis. He had a double-vessel coronary artery bypass graft (CABG) done five years ago. His daily medications included ramipril 5 mg, isosorbide monotrate 30 mg, glymepiride 2 mg, aspirin 75 mg and 2 mg nicoumalone (anticoagulant). On physical examination, he was febrile (102°F) with tachycardia (Heart rate: HR=110/min), blood pressure of 170/86 mm Hg, respiratory rate (RR) 24/min. Jugular venous distension was observed with basal crepitations on chest auscultation. Oxygen 4 litres/min via mask was applied to the patient. Nasogastric and urinary catheterization were done. Intravenous access was secured and isotonic ringers lactate fluid was started. Intravenous tramadol 100 mg was given for analgesia. On admission, haemoglobin was 8.2 gm/ dl, white blood cell (WBC) count was 13,500/*µ*l, blood sugar was 200 mg/dl and international normalized ratio (INR) was 2.34, Arterial blood gas (ABG) revealed PH- 7.37, PCO_2_-35 mm Hg, PO_2_-98mm Hg, HCO_3_- 22 meq/l, BE -2 meq/l, serum potassium- 3.5 meq/l, serum sodium-142meq/l, serum chloride-110 meq/l., Renal and liver function tests were normal. Electrocardiogram revealed HR-134, sinus rhythm, q waves in v1-v4, m-shaped p wave (P mitrale) in lead II. Computed tomography (CT) scan abdomen revealed gall bladder perforation and impacted large gall stone in distal jejunum [Figure 2]. Patient was moved to Intensive Care Unit (ICU) for further management.

Two hours after admission, patient developed pulmonary oedema associated with severe hypertension (180/110 mm Hg), tachycardia (130/min) and tachypnoea (26/ min). Oxygen saturation (SpO_2_) dropped to 58% and a repeat electrocardiogram (ECG) revealed no fresh changes, X-ray chest showed cardiomegaly, pulmonary venous congestion and bilateral alveolar oedema. Cardiac enzymes were within normal range. Patient was intubated after sedation with morphine 4 mg and suxamethonium 100 mg intravenous (IV) and put on controlled ventilation FiO_2_-1.0, subsequently tapered to 0.4. Pulmonary oedema was treated with IV nitroglycerine infusion 10 *µ*g/min, furosemide, blood pressure rise was controlled with IV Hydralazine 20 mg. After local anaesthesia, right internal jugular vein and left radial artery were canulated for central venous and arterial pressure monitoring. Continuous monitoring of ECG, oxygen saturation (SpO_2_) and end-tidal CO_2_ was carried out. Transthoracic echocardiography (TTE) revealed left ventricular ejection fraction (LVEF) of 20-25%, akinetic anterior wall, hypokinetic inferior wall, LV diastolic dysfunction and mitral annular calcification.

On Day 2, X-ray chest showed clear lung fields; blood pressure and HR had stabilized with a SpO_2_ of 99% with FiO_2_ of 0.4 and controlled mode ventilation (CMV) with moderate pressure support. Patient obeyed verbal commands and had stable haemodynamics so was put on synchronized intermittent manual ventilation with pressure support for 4 h to see if weaning off ventilator was possible prior to surgery. 2 units packed cells, 4 fresh frozen plasma and Inj. Vitamin K, 1 mg IV were given to prevent possible intraoperative bleeding from major abdominal procedure. The patient received total of furosemide 200 mg along with Inj. morphine 4 mg, Inj. Tramadol 100 mg for analgesia, Inj. vecuronium (intermittent) and midazolam 2 mg intravenously in the ICU since he was admitted. On Day 2, after a multidisciplinary discussion with the cardiologist, anaesthesiologist and surgeons it was decided to go for the surgery after discussing the risks with the patient’s relatives. Bleeding time done just prior to surgery was 10 min. Patient was given 20 meq/l of potassium correction slowly in fluids intravenously.

Invasive monitoring included arterial pressure and central venous pressures, while non-invasive monitors included continuous cardiac, non-invasive blood pressure, pulse oximeter oxygen saturation and end tidal carbon dioxide (EtCO_2_). The basal parameters in the OT were HR: 138/min, arterial blood pressure (ABP) : 150/70 mm Hg, SpO_2_-98% on CMV (RR-14/min, Tidal volume (TV): 650 ml, FiO_2_ 40%, positive end expiratory pressure (PEEP) 6 mm Hg titrated with arterial blood gas), CVP: 14-16 cm H_2_O, and EtCO_2_: 36-40 mm Hg. After administration of Inj. vecuronium 2 mg and Inj. midazolam 2 mg, TAP block was performed using a 60 ml solution comprising 25 ml 0.5% bupivacaine and 10 ml of 2% lignocaine and 25 ml normal saline.

The TAP block: The triangle of Petit located just anterior to the latissimus dorsi muscle was identified by palpating the iliac crest in an antero-posterior direction, until the edge of latissimus dorsi was felt. The skin was pierced just 3 cm cephalad to the iliac crest over the triangle of Petit with a blunt regional anaesthesia needle (22G, Plexufix^®^, B. Braun, and Melsungen, Germany). Needle was advanced perpendicular to skin in coronal plane till first resistance of external oblique was felt. The first ‘pop’ sensation was felt as the needle gently entered the plane between the external oblique and internal oblique muscle. A second resistance was felt as the needle advanced into the transversus abdominis plane (between internal oblique and transversus abdominis). After careful aspiration to confirm any vascular puncture, 2 ml local anaesthetic was injected initially to open the plane. Further, the needle was advanced intermittently with small injections in a plane parallel to the costal margin pointing towards the iliac crest. A subcostal TAP block was also given after locating the rectus abdominis muscle and feeling its lateral edge and moving a little further beyond the edge, locating the transversus abdominis muscle (which lies just posterior to the rectus muscle superiorly).[2] Fifteen ml local anaesthetic solution was injected each in subcostal TAP and transversus abdominal block (Petit triangle) bilaterally.

Fifteen minutes after the block, patient’s HR was 120/min, ABP: 138/78 mm Hg, CVP: 12 cm H_2_O, EtCO_2_: 40 mm Hg and SpO_2_: 99% with Nitrous oxide in 50% oxygen. Patient was administered propofol sedation bolus dose of 30 mg (0.35mg./kg). Sensory blockade was assessed by observing any rise in HR and/or blood pressure in response to painful stimulus prior to incision. The HR- 128/ min and ABP- 140/80 mm Hg and absence of any movement on forceps stimulation indicated an adequate sensory block. A sensory level from T6-L1 was achieved after 30 min of block procedure. A continuous infusion of propofol 10 mcg./kg/min, midazolam 0.01 mg/kg/h and vecuronium 1 mcg/kg/min was given during three-hour surgery. Resection of the obstructed bowel segment with jejuno-ileal reanastomosis followed by cholecystectomy was performed. The haemodynamics remained stable with HR-110-130/ min and ABP- 120/80-150/98 mm HG intraoperatively.

Intraoperatively, one litre of lost blood volume was replenished with 2 units of whole blood, 4 units of fresh frozen plasma (FFP) and 1.5 litres of crystalloids. Urine output was 2 litres. After the surgery, patient was transferred to ICU, with a CVP of 10-12 cm H_2_O, HR: 106/ min, ABP: 130/70 mm Hg, SpO_2_: 99% and EtCO_2_: 40 mm Hg. His cardiac Doppler done postoperatively showed an LVEF-20% akinetic anterior wall, hypokinetic inferior wall, LV diastolic dysfunction and mitral annular calcification, no clots or vegetations. Intraoperative ABG and electrolyte sample was PH-7.36, PCO_2_-36 mm HG, PO_2_- 112 mm HG, HCO_3_- 22 mm HG BE -2meq/l, potassium 3.8 meq/l, sodium-145 meq/l, chloride 120 meq/l. Pain could not be assessed the traditional way by visual analogue scale as patient was on control mode ventilation for the first postoperative day. At around 2 h after the end of surgery patient opened eyes spontaneously and allowed assessment of sensory level by pinprick. The sensory blockade had receded to T12 in 2 h, and L1 in 4 h post procedure. The total requirement of morphine for analgesia and sedation was Day 1 - 12 mg, Day 2 - 10 mg, and Day 3 -10 mg. Behavioural pain scale[4] was used for pain assessment and sedation was rated with Ramsay sedation scale eight-hourly till extubation on Day 4. On 2^nd^ day, Hb was 10gm%, INR 1.2 and had mildly elevated liver enzymes, though ABG & electrolytes within normal range. Patient did not require ionotropes for cardiac support at any time. On Day 3 he was put on intravenous tramadol 100 mg/tds in addition to morphine, also elastic stockings and enoxaparin 40 mg sc once in a day was started. Day 4, morphine was stopped while patient received only tramadol for analgesia. He was extubated on 4th Postoperative day and switched over to oral medications and discharged on 10^th^ postoperative day. His cardiac Doppler at discharge was the same as preoperative findings (LVEF-20%).

## DISCUSSION

Clinical predictors of perioperative morbidity and mortality like heart failure and risks related to emergency surgery are crucial for deciding a perioperative management plan. We report the successful use of TAP block using Petit triangle and subcostal combined technique for bowel surgery in a high-risk elderly patient.

Though multiple studies have examined the influence of various anaesthetic drugs and techniques of monitoring on cardiac morbidity, it appears that there is no one best myocardial protective anaesthetic technique.[5–8] Opioid-based anaesthesia is popular for cardiovascular stability but is associated with difficulty in weaning from ventilators and known to prolong intensive care stay. All volatile anaesthetics depress myocardial contractility and reduce afterload.[7]

Both general and central neuraxial anaesthesia with varying degree of vasodilatation would be detrimental in a sympathetically driven circulation of cardiac failure as in our patient. High dermatomal blocks specifically pose problems of abrupt hypotension and blockade of cardio accelerator fibres. In a randomized trial of 1021 patients for intraabdominal aortic, gastric, biliary and colon surgeries, there was no overall difference in mortality and morbidity after general anaesthesia/opioid analgesia compared to combined general/epidural anaesthesia and analgesia.[9] Monitored anaesthesia care (MAC) including local anaesthesia and IV sedation/analgesia was associated with increased 30-day mortality in a study by Cohen *et al*.[10] MAC can eliminate some of the general and neuraxial anaesthesia-related complications, but inadequate analgesia, inadvertent vascular injections and systemic toxicity of local anaesthetics is still a possibility.

We decided to administer TAP block for our patient because it provides adequate sensory blockade and effective analgesia during and after the procedure.[2] TAP block could bring down the requirement of other anaesthetic agents used for maintenance of anaesthesia. TAP has been successfully used for laparoscopic surgery.[11]

Abdominal incision and parietal peritoneum (somatic) contribute maximally towards pain of a laparotomy. Terminal branches of T7-T12 and L1 somatic nerves course through the lateral abdominal wall within a plane between the internal oblique and transversus abdominis called as transversus abdominis plane (TAP). The Petit triangle, bounded by the latissimus dorsi posteriorly, external oblique anteriorly and iliac crest at its base is a fixed and easily palpable landmark used to access this neurofascial plane. However, there is some debate over the extent of sensory blockade achieved by TAP block.[2,3] Earlier studies showed a T7-L1 spread of the block after a single posterior TAP injection,[2] while some newer studies revealed that the block failed to spread above T10 so was suitable only for lower abdominal procedures.[12] We decided to administer TAP block with the traditional Petit triangle approach for midline incision as described by McDonnell *et al*., and additional subcostal TAP described by Hebbard et al., for an upper cholecystectomy subcostal incision just parallel to the right subcostal margin about 6-7 inches in length. The sensory blockade in our patient was uniform and persisted for 5 h along the dermatomal level T7-L1. This could be attributed to the combination of TAP and subcostal blocks. The blind technique as used in our patient could have been safer and easier with ultrasound as there is risk of neurovascular injuries with blind techniques.

Tramadol 100 mg was given at admission for analgesia in the patient complaining of abdominal pain. Morphine and midazolam were given just prior to intubation to facilitate intubation without causing any substantial fall in blood pressure like in regular induction with IV anaesthetic agents. The role of morphine was multipurpose. Morphine is known to relieve the dyspnoea associated with acute left ventricular failure and pulmonary oedema from its central sympatholytic and anxiolytic properties.[13] Morphine also worked as a superior analgesic in relieving the abdominal pain significantly reducing the tachycardia and hypertension. Morphine was also used for pain relief in the postoperative period for two days as the analgesic effect of block was expected to wear off. TAP block played a major role in providing analgesia and anaesthesia intraoperatively as propofol was administered in sedation dose of 0.35 mg/kg bolus and later as infusion intraoperatively. Anaesthetic doses of propofol or midazolam or morphine were not used to avoid any exaggerated falls in blood pressure in a cardiac compromised patient (LVEF-20%). Behavioural pain scores involving subscales of facial expression, limb movements and compliance with ventilation (3 no pain-12 maximum pain) of 5-7 on stimulation like endotracheal suction and 3-5 without any stimulation while sedated measured with Ramsay sedation scale 4-6 were recorded first four days post procedure. The pain assessment was limited as readings were taken at fixed time interval of 6 h and may have observer bias as nurses at three duties were involved. Morphine requirement in three days was 32 mg and was tapered off on fourth day. Minimal requirement of morphine during the first 48 h and stable haemodynamic parameters and behavioural pain score of 3-5 at rest and 5-7 at endotracheal suction shows the effectiveness of TAP block.

Apart from the usual beneficial effects of regional anaesthesia technique on postoperative morbidity, TAP block reduces the surgical stress response.[14] Neural blockade techniques can attenuate the classic pituitary, adreno-cortical and sympathetic responses to surgery thereby reducing postoperative organ dysfunction and facilitating early recovery.[15] A sustained or poorly controlled surgical stress response has the potential to cause adverse effects in the perioperative period including pain, cardiac ischaemia, haemodynamic instability, renal decompensation, pulmonary decompensation, increased catabolism, impaired immunity and hypercoagulability syndromes.[16] The sustained release of catecholamines due to unattenuated surgical stress response would result in hypertension, tachycardia, and dysrhythmias may lead to myocardial ischaemia in susceptible patients like this one. Obtundation of surgical stress response with use of TAP block was the key to early and successful recovery of our patient with cardiac compromise.

With regard to doses of local anaesthetics for TAP block, we used 200 mg of lignocaine and 125 mg of bupivacaine, well below the maximum recommended dosages; the potential of systemic toxicity for blocking a large area of abdomen has to be kept in mind. It has been reported earlier that volume of injectate is critical to the success of TAP block and in an average sized adult, 30 ml of local anaesthetic agent can be used for unilateral block and 25-30 ml on each side for a bilateral block.[17] Lignocaine, bupivacaine and ropivacaine have been used so far strictly adhering to maximum recommended dose of each individual agent.[17] TAP block with 40 ml lidocaine 1% (400 mg) was reported to have a potential for systemic toxicity in a study by Naoko Kato et al., with peak concentrations achieved by 30 min of block administration.[18] Few complications reported are liver trauma,[19] intraperitoneal catheter tip and intraperitoneal injection,[20] bowel hematoma[21] and transient femoral nerve palsy.[22]

The decision to withhold or continue anticoagulation should be tailored to meet the best interest of each individual patient after careful review of the risk of thromboembolism off anticoagulation, risk of bleed, and type/duration of the procedure.[23–25] Our patient on aspirin and nicoumalone with an INR 2.34 had intermediate risk of possible thromboembolism but also had risk of bleeding from major abdominal procedure and hence waited for a period of 48 hours. It was advisable to stop aspirin and nicoumalone on admission knowing surgery was imminent and proactive correction of INR with FFP and Vit K is recommended, which we did prior to surgery. The postoperative INR was 1.2. On Day 2 patient was started on enoxaparin 80 mg sc (LMWH) and applied elastic stockings for DVT prophylaxis. TAP block is recommended in patients undergoing abdominal surgery when epidural analgesia is contraindicated.[20] There are no evidence-based randomized trials comparing epidural analgesia and TAP block so far.

In summary, the present case report shows that TAP block can be effectively used to provide adequate analgesia for abdominal surgeries, especially in patients with compromised physiology. Ultrasound-guided TAP block holds potential for a safer and effective block in future practice. Future studies are necessary so as to fully understand its potential as an analgesic, part of multimodal analgesic regimen and an anaesthetic purpose with clear dose-response relationship and segments of nerve block achieved.
